# Pharmacological activation of pyruvate kinase M2 reprograms glycolysis leading to TXNIP depletion and AMPK activation in breast cancer cells

**DOI:** 10.1186/s40170-021-00239-8

**Published:** 2021-01-22

**Authors:** Fadi Almouhanna, Biljana Blagojevic, Suzan Can, Ali Ghanem, Stefan Wölfl

**Affiliations:** Institute of Pharmacy and Molecular Biotechnology, Heidelberg University, Im Neuenheimer Feld 364, 69120 Heidelberg, Germany

**Keywords:** Pyruvate kinase M2, AMPK, TXNIP, Glycolysis, Cancer metabolism, Breast cancer

## Abstract

**Background:**

Aerobic glycolysis, discovered by Otto Warburg, is a hallmark of cancer metabolism even though not yet fully understood. The low activity of the cancerous pyruvate kinase isozyme (M2) is thought to play an important role by facilitating the conversion of glycolytic intermediates to other anabolic pathways to support tumors’ high proliferation rate.

**Methods:**

Five breast cancer cell lines representing different molecular subtypes were used in this study where real time measurements of cellular bioenergetics and immunoblotting analysis of energy- and nutrient-sensing pathways were employed to investigate the potential effects of PKM2 allosteric activator (DASA-58) in glucose rewiring.

**Results:**

In this study, we show that DASA-58 can induce pyruvate kinase activity in breast cancer cells without affecting the overall cell survival. The drug is also able to reduce TXNIP levels (an intracellular glucose sensor) probably through depletion of upstream glycolytic metabolites and independent of AMPK and ER signaling. AMPK shows an induction in phosphorylation (T172) in response to treatment an effect that can be potentiated by combining DASA-58 with other metabolic inhibitors.

**Conclusions:**

Altogether, the multifaceted metabolic reprogramming induced by DASA-58 in breast cancer cells increases their susceptibility to other therapeutics suggesting the suitability of the intracellular glucose sensor TXNIP as a marker of PK activity.

**Supplementary Information:**

The online version contains supplementary material available at 10.1186/s40170-021-00239-8.

## Background

Aberrant metabolism is one of the hallmarks of cancer [1, 2]. The Warburg effect (aerobic glycolysis) is among the first identified metabolic alterations of cancer cells [1]. Despite the low efficiency of glycolysis in terms of ATP production in comparison to oxidative phosphorylation, many cancers adopt the glycolytic phenotype [3]. This indicates that the elevated glycolysis confers other benefits to cancer cells than replenishing the ATP pools, as the upregulated glycolysis seems to help cancer cells to cope with their high proliferation rate [4]. Although not yet fully understood, many theories have emerged trying to understand the benefits of the Warburg effect to cancers, i.e., the elevated glycolysis rate is suggested to help accumulate more upstream glycolytic intermediates to be used for anabolic pathways branching from the main glycolytic stream [5]. The pentose phosphate pathway (PPP) [6] and the serine synthesis pathway (SSP) [7] are examples of the anabolic pathways branching from glycolysis and reported to support cancer proliferation. The altered expression of glycolytic enzymes drives the tumors’ deviated glycolysis supporting growth [3]. Pyruvate kinase (PK) is a key glycolytic enzyme that is involved in the altered metabolic phenotypes of many cancers [8]. More precisely, cancer cells preferentially splice pre-mRNA of the PKM gene to produce PKM2 rather than PKM1, the former possesses the ability to finely tune its activity in response to nutrient availability and to other different cellular signals [9]. Unlike PKM1, the M2 isotype exists in two conformations: a fully active tetramer and a less active dimer whereas different post translational modifications dictate the dimer/tetramer ratio and thus the activity of the enzyme [10]. The low affinity of the dimer to its phosphoenolpyruvate (PEP) substrate helps cancer cells to cope with the high levels of reactive oxygen species (ROS) by inducing the flow of glucose 6-phosphate through the PPP, producing the reducing equivalent NADPH [11]. Furthermore, the expression of PKM2 was associated with non-metabolic benefits to cancers, by translocating into the nucleus; PKM2 exerts a protein kinase and a transcriptional co-activator role promoting tumorigenesis through the STAT3/PKM2/Hif1**α** feedback loop [12]. The aforementioned reasons propose the importance of a strict control of glycolysis in cancer cells and the ability to tune glycolytic metabolites based on the cellular needs. Another level of regulating PKM2 activity is mediated by the availability of serine–serine shown to enhance the affinity of PKM2 to PEP, and thus, under serine abundant conditions, PKM2 is “active,” but when serine is scarce PKM2 activity drops allowing for upstream metabolites to be shunted into the serine biosynthesis pathway [13].

Cancer cells—for example—are sensitive to low energy status [14] that is usually depicted by elevated AMP/ADP to ATP ratio and translated into phosphorylation and activation of AMP-activated protein kinase (AMPK) [15]. The activated AMPK reduces anabolism and induces catabolism aiming to re-equilibrate ATP levels [14, 16]. Drugs activating AMPK are not only essential in treating metabolic diseases (metformin and diabetes), but also studied for their potential anticancer effects [17, 18]. Moreover, AMPK activation is also reported to inhibit mTOR signaling under low energy status and thus playing a cytostatic effect [19].

Replacing PKM2 by another PK isozyme with higher affinity to PEP was assessed as a potential way to hamper the benefits of PKM2 in cancers [10]. The proposed pro-cancer roles of PKM2 lead to the discovery of specific and allosteric PKM2 activators, e.g., DASA-58 and TEPP-46, as both compounds showed an enhanced pyruvate kinase (PK) activity only in cells expressing PKM2 while exhibiting no or very low activity in case of PKM1 or other PK isozymes [20]. PKM2 pharmacological activation was shown to potentiate the anticancer effects of the glycolytic inhibitor 2-deoxyglucose (2DG) in cancer cells from different tissue origin [21]. Besides, PKM2 deletion was shown to increase the formation of tumors in a mouse model of breast cancer [22].

In this study, we used five breast cancer cell lines representing different molecular subtypes to investigate the response to PKM2 pharmacological activation in the light of the basal activity levels represented by each of the cell lines included in the study. Furthermore, we aimed to investigate the possible intracellular glucose rewiring under PKM2 activation and whether this activation is changing the energy homeostasis in these cells.

## Methods

### Cell culture

All cell lines used in this study (MDA MB 231, MDA MB 468, HCC 1443, T47-D, MCF7, LnCap, PC3, and DU145) were kept in a humidified cell culture incubator at 37 °C with 5% CO_2_. Cells were also culture in Dulbecco’s Modified Eagle Medium (DMEM)-GlutaMAX™ (Gibco, Germany), supplemented with 10% FCS (v/v) (Gibco, Germany), 1% penicillin/streptomycin (v/v) (Gibco, Germany). MDA MB 468, HCC 1443, and T47-D are kind gifts from Dr. Stefan Wiemann (DKFZ, Heidelberg). Indicated drugs were added to the standard culture medium for the indicated time points. PKM2 allosteric activators DASA-58 and TEPP-46 (Cayman Chemical-Biomol GmbH, Germany), dehydroepiandrosterone (DHEA), CBR-5884, AZD-3965, metformin, and sodium oxamate (Cayman Chemical-Biomol GmbH, Germany) 2-deoxyglucose (Fluka-Sigma-Aldrich, Germany) Rotenon, carbonyl cyanide 3-chlorophenylhydrazone (CCCP), tamoxifen, oligomycin, and sodium dichloroacetate (Sigma Aldrich, Germany).

### Mitochondrial membrane potential and glucose uptake measurements using FACs

Cells were seeded at a density of 50,000 cells/well in 12 well-plate and treated with the indicated drug or the appropriate mock control for the indicated time points. For mitochondrial membrane potential (MMP), cells were rinsed with PBS, harvested, and incubated with 2 μM JC1 (30 min) (Sigma Aldrich, Germany). After the incubation period, cells were collected and re-suspended in fresh phenol red-free DMEM and analyzed with FACs. For glucose uptake assay, 50 μM fluorescently labeled glucose analog 2-[N-(7-nitrobenz-2-oxa-1,3-diazol-4-yl) amino]-2-deoxy-D-glucose (2-NBDG) (Cayman Chemical- Biomol GmbH, Germany) was added to each well 2 h before the end of the treatment period and kept in the incubator. Subsequently, cells were washed once with PBS, harvested, re-suspended in phenol-red free DMEM, and analyzed with FACs. FACs Guava instrument (Merck, Germany) and Guavasoft 3.1.1 (Merck, Germany) were used for the FACs analysis.

### Online measurements of extracellular acidification and oxygen consumption using PreSens Biosensor Dish Reader (SDR)

Cells were seeded at a concentration of 150,000 cells/well in OxoDish and HydroDish 24-well plates (PreSens Precision Sensing GmbH, Regensburg, Germany) and incubated in standard conditions. As described previously [23], fluorescence-based sensors embedded at the bottom of each well could be read continuously using SensorDish Readers (SDRs) placed inside a standard incubator. The indicated treatments were added to each well; subsequently, the plates were mounted on readers inside the standard incubator, and medium pH values/oxygen saturation values were recorded each 10 min.

### Isolation of mouse-liver mitochondria

Mitochondria were isolated according to described procedures with minor modifications [24, 25]. Mouse (wildtype, C57BL/6) liver mitochondria were isolated by Dounce homogenization and differential centrifugation in isolation buffer (300 mM trehalose, 10 mM HEPES-KOH pH 7.7, 10 mM KCl, 1 mM EGTA, 1 mM EDTA, 0.1% fatty acid free BSA). The homogenate was centrifuged for 5 min at 1000 *g*, 4 °C. The supernatant was collected and centrifuged for 2 min at 15,800 *g*, 4 °C. The mitochondrial pellet was re-suspended in a small volume of isolation buffer, and the last centrifugation step was repeated. The amount of total protein was measured using Bradford assay.

### Measurement of mitochondrial oxygen consumption

Oxygen consumption by intact mitochondria was measured using the OxoPlate sensor system (PreSens, Regensburg, Germany) in a 96-well plate format. Fluorescence was measured in dual mode, excitation 540 nm and emission 650 nm, with reference emission 590 nm. Signal ratio 650 nm/590 nm corresponds to the oxygen partial pressure. Eleven micrograms of freshly isolated mitochondria was suspended in 100 μL of respiration buffer (25 mM sucrose, 100 mM KCl, 75 mM mannitol, 5 mM MgCl2, 10 mM KH_2_PO_4_, 0.5 mM EDTA, 10 mM TRIS, 0.1% fatty acid-free BSA, pH 7.4) containing 10 mM pyruvate, 2 mM malate, 2 mM ADP, 0.5 mM ATP, and the respective test compound. Kinetic fluorescence measurement was performed for 400 min with 5 min intervals using a Tecan Safire 2 reader (Tecan, Maennedorf, Switzerland) at 37 °C. A breathable membrane (Diversified Biotech, Dedham, MA, USA) was used to seal the plate during signal detection. 5 µM rotenone (inhibitor of respiratory chain complex I) and 5 μM oligomycin (inhibitor of mitochondrial ATP synthesis) served as controls.

### Western blotting

After treatment and at the indicated time, cells were washed once with PBS and lysed with 6 M urea buffer equipped with protease and phosphatase inhibitors (PMSF, aprotinin, pepstatin, leupeptin, sodium orthovanadate, and sodium pyrophosphate). Protein concentration was measured using Bradford reagent (Sigma-Aldrich, Germany). Equal amounts of protein (50 μg) were loaded into each well of 10% SDS-PAGE for resolving the samples. Subsequently, separated proteins were blotted onto a PVDF membrane (GE Healthcare, Germany). Membranes were then washed with TBS/tween and blocked for 3 h at room temperature with fat-free dried milk before incubation with the indicated primary antibodies at 4 °C for overnight.

Anti-β-actin and anti-vinculin antibodies were purchased from Santa Cruz Biotechnology, whereas anti-mouse and rabbit IgG horseradish peroxidase (HRP)-linked antibodies were purchased from Cell Signaling Technology.

Membranes were developed using Western Lightning^TM^ Plus-ECl (Perkin Elmer, Germany) as HRP substrate. Proteins of interest were detected using a Fujifilm LAS-3000 imaging system.

### RNA interference

Small interfering (si)RNA against AMPKα1, STAT3, LKB1, and negative control (NC) (Table 1) were synthesized by Riboxx (Riboxx GmbH, Radebeul, Germany). Fifty picomoles of the respective siRNA/negative control were diluted in 100 μL Opti-MEM Reduced Serum Medium (Gibco, Germany) and added to each well of 24-well plate then complexed with 1 μL transfection reagent Lipofectamine® 3000 (Thermo Fischer, Germany). Fifty thousand cells/500 μL were added to each well, mixed gently, and incubated in standard incubator. On the following day, the respective treatments were performed as described.

**Table 1:** The sequences of the siRNAs used in the study

Target	Sense	Anti-sense
NC	UUGUACUACACAAAAGUACCCCC	GGGGGUACUUUUGUGUAGUACAA
AMPKα1	GGGGGCCACAAUCAAAGAUAU GGGGGAUUGGAACAUGAUUUA GGGGGAGAGCUAUUUGAUUAUA	AUAUCUUUGAUUGUGGCCCCC UAAAUCAUGUUCCAAUCCCCC UAUAAUCAAAUAGCUCUCCCCC
LKB1	GGCUCUUACGGCAAGGUGA	UCACCUUGCCGUAAGAGCC
STAT3	CGTCATTAGCAGAATCTCATT	TGAGATTCTGCTAATGACGTT

### Enzymatic activity

Cells were cultured as described before [26] until they are 60–70% confluent, subsequently treated with DASA-58 (15 μM), and lysed at the indicated time point. Cells were collected washed with PBS and re-suspended in ice-cold lysis buffer (KCl, MgCl2, HEPES, EDTA). Cells were lysed by shaking with acid-washed glass beads in a bead mill for 30 s. For pyruvate kinase activity, equal amounts of protein were added to each reaction mix (500 ng), and decreased absorbance at 340 nm was monitored for 20 min at 30 °C using Tecan plate reader (Tecan, Germany). The slopes were calculated from the initial linear range. Pyruvate kinase activity reaction mix contains 47 mM potassium phosphate, 6.7 mM MgSO4, 0.44 mM NADH, 1.5 mM ADP, 0.3 mM PEP, and 1 unit of lactate dehydrogenase. The drop in absorbance at 340 nm resulting from the oxidation of NADH was monitored kinetically and used to calculate the activity of the enzyme.

For G6PD activity, 1000 ng of native lysate was added to either reaction mix 1 containing 2 mM glucose-6-phosphate, 1 mM NADP^+^, 50 mM TRIS/HCl buffer, pH 8.2, 100 mM KCl, and 5 mM MgSO_4_ or to reaction mix 2 containing 2 mM 6-phosphogluconate instead of glucose-6-phosphate reaching 100 μL final volume. The increase in absorbance at 340 nm resulted from the buildup of NADPH was monitored kinetically for 120 min at 30 °C using a Tecan plate reader (Tecan, Germany).

Both reactions (reaction mix 1 and 2) were run in parallel, and the increase in absorbance resulting from G6PD activity was calculated by subtracting the absorbance values of reaction 2 from reaction 1.

### Sulforhodamin-B (SRB) assay

Cells were cultured in 96-well plate at a density of 5000 cell/well, 24 h after seeding medium was removed and replaced with fresh medium containing the indicated treatment. At the end of the treatment period, cells were fixed with 10% trichloroacetic acid for 1 h at 4 °C; subsequently, the plated were washed twice with water and dried. One hundred microliters of 0.054 sulforhodamine B solution in acetic acid (sulforhodamine powder Sigma Aldrich Germany) was added to each well and incubated for 30 min at RT. Excess dye was washed twice with 1% acetic acid. After drying the plates, the dye was solubilized in 200 μL 10% tris buffer (pH 10.5), and the absorbance was measured at 535 nm using a Tecan ultra plate reader (Tecan, Germany).

### Cell Viability assay

Cells were seeded in 48-well plate at a density of 10,000 cells/well; on the following day, the medium was replaced with fresh medium containing the indicated treatment. Seventy-two hours post-treatment, the medium was replaced with DMEM (1% FCS) containing 0.5 g/mL 3-(4,5-Dimethylthiazol-2-yl)-2,5-diphenyl-2H-tetrazolium bromide (MTT) (Sigma Aldrich, Germany) and cells incubated for 2 h. Subsequently, the intracellular formazan was dissolved in DMSO, and the absorbance at 595 was measured using a Tecan ultra plate reader (Tecan, Germany).

### Lactate measurement

Cells were seeded in 96-well plate at a density of 5000 cells/well; 24 h later, the medium was replaced with fresh medium containing either DASA-58 (15 μM), or TEPP-46 (30 μM), or the DMSO (mock). Cells were incubated with the treatment for 72 h; subsequently, the concentration of extracellular lactate was measured using the Lactate-Glo^TM^ assay kit (Promega, Germany) according to the manufacturer’s protocol. After using the supernatant to measure lactate concentration, cells were fixed and stained with SRB (refer to 2.8.) to serve as control of cell content. All lactate measurements were normalized to total protein measured with SRB assay.

### Statistical analysis

Data analysis was performed using Microsoft excel and GraphPad Prism. Data were plotted as mean values with error bars representing standard deviation. ImageJ was used for densitometric analysis of western blot images. For enzymatic activity, the slopes of the graphs were calculated from the initial linear range and plotted as fold change. Two-tailed Student’s *t* test was used to calculate statistical significance between measured parameters. *P* values equal to or smaller than 0.05, 0.01, and 0.001 are depicted as *, **, and *** respectively.

## Results

### Basal PKM2 expression and activity levels in different BCa cells influence the cellular response to PKM2 pharmacological activation

**Fig. 1:**
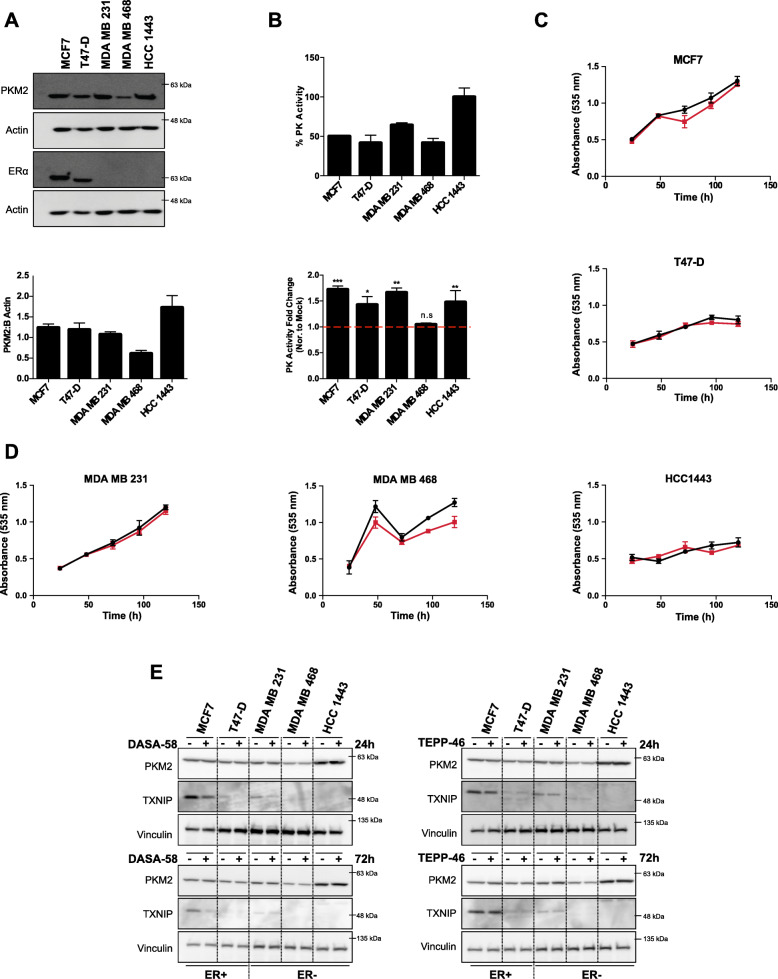
PKM2 pharmacological activation enhances pyruvate kinase activity in breast cancer cells without a clear effect on proliferation. **a** Representative western blot showing different PKM2 expression levels in five breast cancer cell lines (upper panel) with a densitometric analysis showing levels of PKM2 normalized to actin values (lower panel). **b** PK basal activity in BCa cells represented as percentage PK activity (PK activity in HCC1443 is considered 100%) measured in crude cell extracts (upper panel), PK activity levels in response to DASA-58 (15 μM), data shown as fold change in activity normalized to mock treatment, all tested cell lines except MDA MB 468 respond to DASA-58 treatment by increasing PK activity as measured in crude cell extracts (lower panel). **c**, **d** Total protein staining using SRB assay showing no significant change in cell survival in response to DASA-58 (15 μM; red line) in comparison to mock treatment (black line) in ER+ BCa cells (**c**) and ER − BCa cells (**d**) for up to 5 days under standard culture conditions. **e** Western blots showing the expression of PKM2 and TXNIP in response to either DASA-58 (15 μM) or TEPP-46 (30 μM) in short (24 h) and long (72 h) treatments. PKM2 activators do not change PKM2 levels in the tested cell lines but seemingly reduce TXNIP levels in cells expressing detectable TXNIP levels

We started our study by investigating the protein as well as the activity levels of pyruvate kinase M2 in a panel of estrogen receptor (ER) positive (+) and negative (−) breast cancer (BCa) cells. Protein extracts were analyzed with SDS-PAGE, whereas all studied cell lines showed comparable PKM2 protein levels, except HCC1443 cells and MDA MB 468 (Fig. 1a) that showed the highest and lowest PKM2 protein levels, respectively. We then compared the basal PK activity levels in all the studied cell lines, and as shown in Fig. 1b, PK activity was the highest in HCC1443 cells and the lowest in MDA MB 468 cells corresponding to the expression levels of PKM2. PK activity levels were different with ER+ cells (MCF7 and T47-D) showing a lower activity level in comparison to triple negative cells (MDA MB 231 and HCC 1443) which showed an elevated PK activity with an exception of MDA MB 468 where activity levels are comparable to ER+ cells. It is also worth mentioning that PK activity was significantly different between MCF7 and MDA MB 231, for example, even though PKM2 levels were comparable between these two cell lines. We also assessed the cellular response to the PKM2 allosteric activator DASA-58 on PK activity and on proliferation. All studied cell lines responded to the treatment by showing a significant increase in PK activity (measured in cell crude lysates) except MDA MB 468, the cell line with the lowest PK activity and PKM2 level (Fig. 1b). The possible toxic effects of the PKM2 activator were tested using sulforrhodamine B (SRB) assay for a panel of BCa cell lines. DASA-58 as shown in Fig. 1 c and d did not have any significant impact on the cellular proliferation as all the five investigated BCa cell lines showed no significant change in cell number (measured with SRB) for up to 5 days under normal culture conditions.

DASA-58 and TEPP-46 (both PKM2 activators with distinct chemical structure) were used to exclude structure-related off-target effects. All studied cells show no change in PKM2 expression in response to either DASA-58 or TEPP-46 in short-term (24 h) and long-term (72 h) treatment (Fig. 1e). Furthermore, and in order to check if PKM2 activation is affecting the cellular nutrient sensing mechanism, we probed with an antibody against the thioredoxin-interacting protein (TXNIP), a protein reported to sense intracellular levels of the upstream glycolytic metabolites [27]. The cells showing detectable levels of TXNIP (MCF7 and MDA MB 231) exhibited a strong reduction in TXNIP levels in response to 24 h treatment with DASA-58; nevertheless, this effect was less pronounced in 72 h treatment in case of MDA MB 231. On the other hand, TEPP-46 treatment showed reduced TXNIP levels only in case of short treatment (24 h) and in MCF7 cells (Fig. 1e).

These data confirm the efficacy of PKM2 activation on increasing PK activity levels in BCa cells without affecting the overall cellular survival. Additionally, we sought of further investigating the effects of PKM2 activation on cellular bioenergetics.

### PKM2 activation increases extracellular acidification and lactate levels in different types of BCa cells

**Fig. 2:**
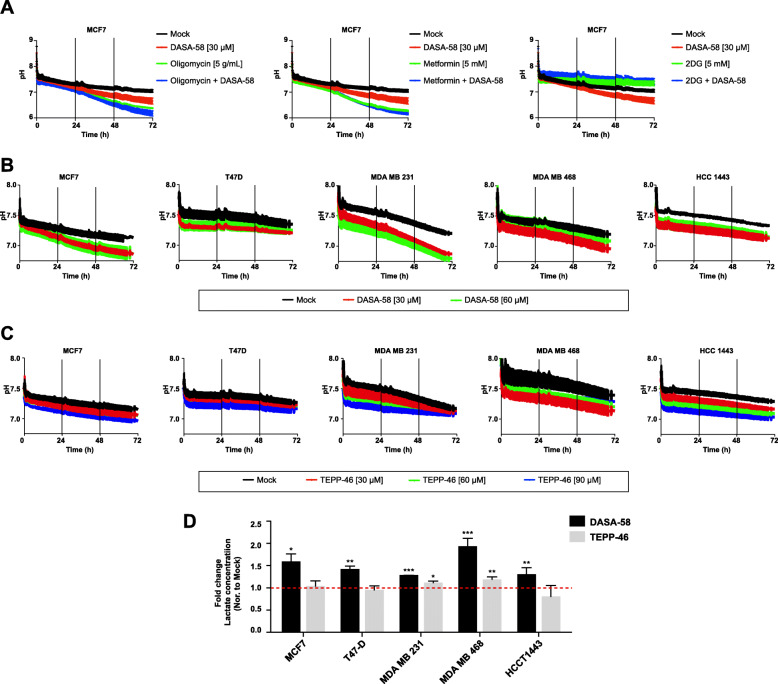
DASA-58 treatment enhances extracellular acidification and lactate levels in BCa cell lines. **a**–**c** Online measurement of extracellular acidification using pH sensors embedded on the bottom of 24-well plates in MCF7 cells in response to DASA-58 (30 μM) alone or combined with either the ATP-synthase inhibitor oligomycin (5 μM), the mitochondrial complex one inhibitor metformin (5 mM), or the glycolytic inhibitor 2DG (5 mM) (**a**). Or in five different BCa cell lines in response to DASA-58 (30 μM and 60 μM) (**b**) or to TEPP-46 (30 μM, 60 μM, and 90 μM) **c**. **d** Fold change in extracellular lactate levels in five BCa cells lines in response to DASA-58 (30 μM) or to TEPP-46 (30 μM)

After confirming the ability of DASA-58 to increase PK activity in BCa cells, we moved to assess whether the treatment is affecting lactate production and secretion. Figure 2 shows extracellular acidification levels corresponding to cellular lactate production. Increasing concentrations of either DASA-58 or TEPP-46 reduced extracellular pH in all the studied celllines (Fig. 2 b and c). This induction is clearly visible after a long incubation period. To further confirm that the drop in pH value is due to enhanced glycolytic flow, we treated MCF7 cells with either the glycolytic inhibitor (2DG) alone or in combination with DASA-58 which reduced medium acidification in the presence and absence of DASA-58 (Fig. 2a). Another feature exhibited by MCF7 cells is their ability to boost glycolysis depicted by strong reduction in pH values (enhanced lactate production) when treated with the ATP synthase inhibitor oligomycin (Fig. 2a) or with the mitochondrial complex I inhibitor metformin (Fig. 2a). To further validate the monitored increase in extracellular acidification, we measured lactate content in the medium in response to both PKM2 activators. As shown in Fig. 2d, DASA-58 is capable of increasing lactate concentration in the medium in all studied cells lines while TEPP-46 at the used concentration is only capable of significantly inducing lactate levels in MDA MB 231 and MDA MB 468. However, the higher potency of DASA-58 in inducing medium lactate levels in comparison to TEPP-46 could be related to the concentrations used in this study.

Moreover, to validate these findings in a different cellular background, we performed additional experiments in a panel of prostate cancer cell lines. DASA-58 and TEPP-46 were capable of inducing extracellular acidification levels in all analyzed prostate cancer cell lines (supplementary figure S1).

### PKM2 activation decreases oxygen consumption without damaging the mitochondria

**Fig. 3:**
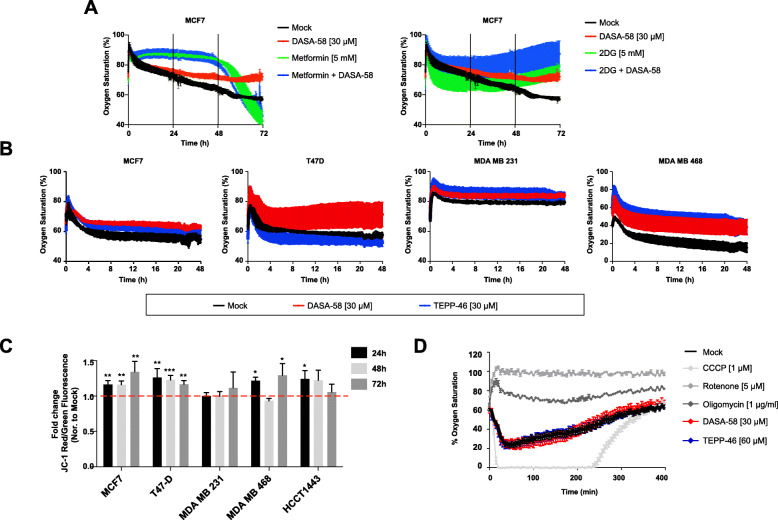
DASA-58 affects respiration levels in BCa cells without an indication of mitochondrial damage. **a**, **b** Online measurement of oxygen saturation in culture medium using oxygen sensors embedded in 24-well plates in MCF7 (metformin and 2DG were used as controls) (**a**) and in four different BCa cell lines in response to either DASA-58 (30 μM) or TEPP-46 (30 μM) (**b**). **c** Mitochondrial membrane potential measured with JC-1 dye in different BCa cell lines in response to DASA-58 (15 μM) showing a slight increase or no change in membrane potential in all tested cell lines and up to 72 h of treatment (data presented as red/green fluorescence fold change in comparison to mock treatment). **d** Online oxygen consumption in isolated mouse liver mitochondria using oxygen sensors embedded in 96-well plate showing no direct effect of neither DASA-58 (30 μM) nor TEPP-46 (60 μM) on mitochondrial activity, 5 μM of rotenone (complex I inhibitor) and 1µg/mL of oligomycin (ATP synthesis inhibitor) are used as control

To further investigate the metabolic shift induced by PKM2 pharmacological activation, we analyzed oxygen saturation in culture medium. Our pH measurement showed that increased PKM2 activity was leading to reduction in medium pH in BCa cells. We therefore sought to investigate mitochondrial activity in response to DASA-58 treatment. Figure 3 a and b show oxygen saturation levels in culture medium in response to DASA-58. In Fig. 3 a, we see higher oxygen saturation levels (%) in response to the DASA-58 in MCF7 cells indicating a lower oxygen consumption. Interestingly, the reduction in oxygen consumption is also observed in the other studied BCa cell lines (Fig. 3b). Furthermore, both used pharmacological activators induce similar effects in terms of medium oxygen saturation (Fig. 3b) even though TEPP-46 seems to be less potent (in case of T47-D for example).

The effects of DASA-58 treatment on the mitochondrial inner membrane potential (MMP) are measured with the formation of red JC-1 aggregates (Fig. 3c). PKM2 activation and as shown in Fig. 3c causes a slight but significant increase in MMP levels, an affect that is more pronounced in MCF7 and T47-D cells. MDA MB 231 cells for example do not show any change in MMP values in response to DASA-58 treatment, while MDA MB 468 and HCC1443 cells also show a slight induction in MMP levels, which are significant in case of MDA MB 468 after 24 and 72 h treatment, and at 24 h in case of HCC1443.

To further confirm that DASA-58 or TEPP-46 does not have a direct inhibitory role on mitochondria, we treated mitochondria isolated from mouse liver with either of the PKM2 activators and measured oxygen saturation in medium over time. As shown in Fig. 3 d, neither of the used drugs inhibited oxygen consumption when tested on isolated mitochondria. Thus, the reduced oxygen consumption exhibited by MCF7 cells under DASA-58 treatment is not due to any possible direct mitochondrial inhibition.

Furthermore, different blockers of glycolysis’ branching pathways (Fig. 6a and b) tested in combination with DASA-58 showed enhanced anti-proliferation effects when DASA-58 is combined with the G6PD inhibitor DHEA, PGDH inhibitor CBR-5884, and MCT inhibitor AZD-3965. Moreover, locking PKM2 in an active state using DASA-58 enhanced the antitumor effects of different metabolic stressors and inhibitors of glycolysis’ branching pathways (2DG, CCCP, DHEA, CBR-5884, and AZD-3965). These observations could be exploited aiming to open a new therapeutic window by using PKM2 activators to potentiate the effects of other metabolic inhibitors and ROS inducing agents.

Altogether, our data suggests that enhanced lactate production caused by DASA-58 treatment is associated with decreased oxygen consumption without a clear sign of damaged mitochondria.

### PKM2 activation leads to AMPK signaling activation and TXNIP depletion

**Fig. 4:**
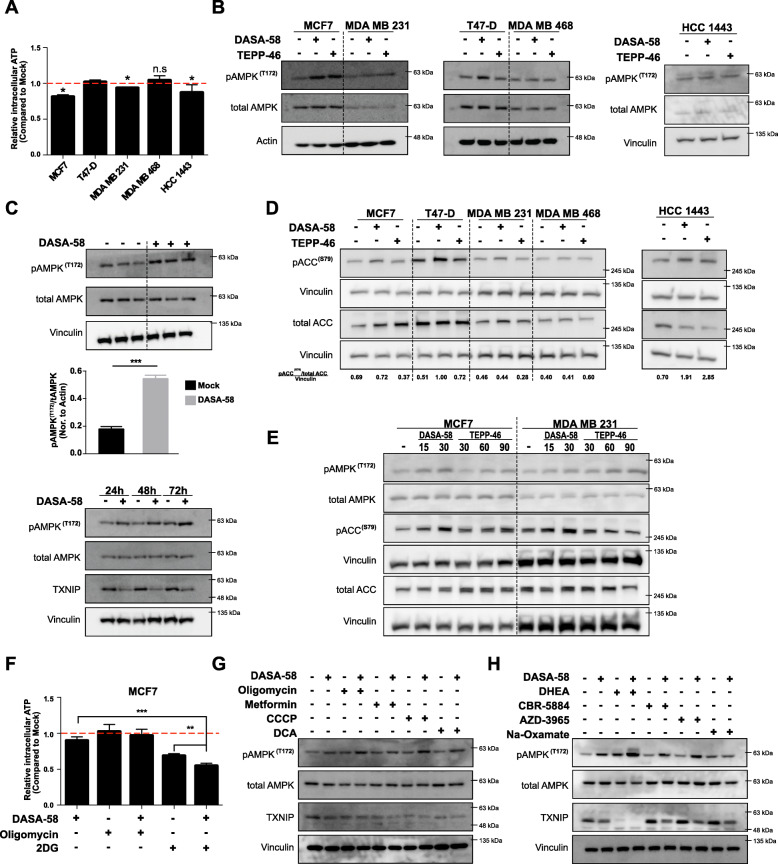
PKM2 activation reduces ATP pools leading to AMPK activation and energy crisis. **a** Cellular ATP levels in response to 72 h DASA-58 (15 μM) treatment in different BCa cell lines showing slight reduction in ATP levels in MCF7, MDA MB 231, and HCC1443 cell lines. **b** Western blot analysis of different BCa cell lines in response to 72 h treatment of either DASA-58 (15 μM) or TEPP-46 (30 μM), showing levels of pAMPK (T172) and total AMPK. **c** Western blot showing the induction in pAMPK (T172) levels in MCF7 cells in response to 72 h DASA-58 (15 μM) treatment in triplicates (upper panel), with the corresponding densitometric analysis representing pAMPK/tAMPK as fold change normalized to actin levels, and the induction of pAMPK (T172) levels in MCF7 cells in response to DASA-58 (15 μM) at 24, 48, and 72 h of treatment (lower panel). **d** Western blot analysis of different BCa cell lines showing the expression of pACC, tACC, and vinculin in response to either DASA/58 (15 μM) or TEPP-46 (30 μM); numbers underneath the figure represent the densitometric analysis of pACC/tACC band intensities normalized to the corresponding vinculin bands. **e** Western blot analysis showing the levels of pAMPK/tAMPK and of pACC/tACC together with vinculin in response to increasing concentrations of DASA-58 and TEPP-46 in MCF7 and MDA MB 231. **f** Relative ATP levels in MCF7 in response to DASA-58 (15 μM) alone or combined with oligomycin (5 μM) or 2DG (5 mM). **g**, h Western blots with pAMPK (T172) and TXNIP levels in response to either DASA-58 (15 μM) alone or combined with mitochondrial modulators (**g**) or glycolytic branching/related pathways modulators (**h**) showing that none of the used combo treatments is able to rescue TXNIP levels in the presence of DASA-58 and that mitochondrial inhibitors enhance PKM2 effects on activating AMPK signaling (depicted as T172 phosphorylation of AMPK)

We next sought to investigate the cellular energy state in response to DASA-58 treatment. Figure 4a shows a slight decrease in intracellular ATP levels that is yet significant in MCF7, MDA MB 231, and HCC1443 cells in response to 72 h of DASA-58 treatment. Furthermore, and as shown in Fig. 4b and c, the energy crisis is translated into AMPK activation (depicted by increased AMPK phosphorylation at T172) in all studied cells except MDA MB 468 that did not show any change in AMPK phosphorylation status in response to the treatment. Figure 4b and c also show that both DASA-58 and TEPP-46 have similar effects on AMPK phosphorylation at T172 with MCF7 cells being the most sensitive cell line to the treatment. A time course treatment of DASA-58 in MCF7 cells shows increased T172 phosphorylation of AMPK accompanied by decreased TXNIP levels in all studied time points (Fig. 4c). We later investigated the phosphorylation levels of acetyl-CoA carboxylase (ACC) downstream of AMPK, and as shown in Fig. 4d an induction in the phosphorylation of ACC (S79) was only present in case of HCC1443 and in case of T47-D with DASA-58. In Fig. 4e, a concentration gradient treatment with both activators showed that increased concentration of DASA-58 and TEPP-46 is capable of inducing the phosphorylation status of AMPK and its downstream effector ACC in MCF7 and MDA MB 231 cells. These effects were also validated in a panel of prostate cancer cell lines (LnCap, DU145, and PC3). Supplementary figure S2 shows that only DASA-58 was capable to significantly induce the levels of phospho-ACC (S79) while TEPP-46 failed to significantly change ACC phosphorylation in the used concentration (30 mM).

We further investigated the intracellular ATP levels in MCF7 in response to either 2DG or oligomycin as single treatments or combined with DASA-58, as shown in Fig. 4f. Oligomycin treatment does not cause any measurable reduction in ATP levels but rather rescues the reduction caused by DASA-58. The induced glycolysis (depicted in medium acidification) in response to oligomycin is apparently adequate to stabilize cellular ATP pool when mitochondrial ATP synthesis is inhibited. 2DG treatment depleted ATP levels in MCF7 cells (Fig. 4f). Moreover, 2DG and DASA-58 combination further decreased ATP levels (Fig. 4f). We further analyzed the phosphorylation status of AMPK and the expression of TXNIP (intracellular glucose sensor) in response to DASA-58 combined with a wide range of metabolic inhibitors. Figure 4 g shows that oligomycin, CCCP, and DCA but not metformin further potentiates the effects of PKM2 activation on AMPK phosphorylation status in MCF7 cells when added to the conditioned medium 24 h before the end of the treatment period (72 h). These combinational treatments failed also to rescue the TXNIP expression in response to DASA-58 treatment. To test the hypothesis that PKM2 activation enhances the sensitivity to other drugs blocking anabolic pathways branching from glycolysis, we tested the effects of combining DASA-58 with DHEA (G6PD inh.), CBR-5884 (serine biosynthesis inh.), AZD-3965 (MCT inh.), and sodium oxamate (lactate dehydrogenase inh.). Figure 4 h shows that only the G6PD inhibitor DHEA potentiates the effect of DASA-58 on AMPK activation while the other combi regimens have no or minor role. Interestingly, Fig. 4 h shows that the reduction in TXNIP expression (indicative low concentrations of upstream glycolytic intermediates) could not be rescued with any of the combination treatments.

Taken together, the monitored alterations in glycolytic/respiration rate in BCa cells in response to DASA-58 treatment are translated into a cellular energy crisis leading to AMPK signaling activation and interestingly reduced TXNIP levels. TXNIP is induced in the cell in response to elevated levels of glucose-6-phsophate and other glycolytic intermediates after the formation of the heterodimeric transcription factor MondoA/MLX that binds the TXNIP promoter and increases its expression [27].

### TXNIP reduction is independent of AMPK signaling and probably due to upstream glycolytic metabolites depletion

**Fig. 5:**
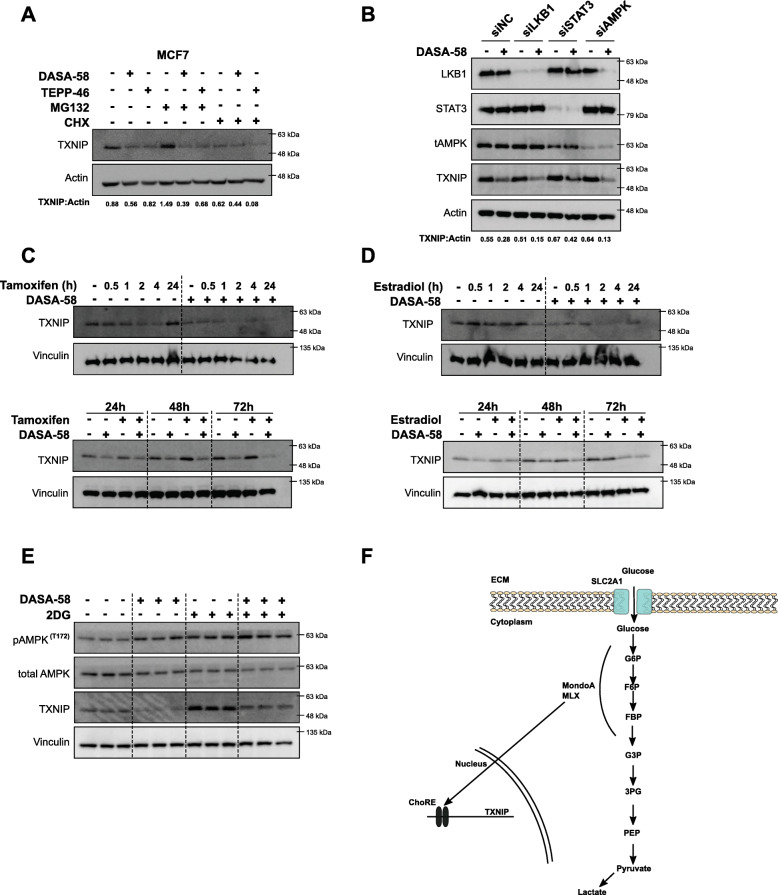
TXNIP reduction in response to PKM2 activation is probably due to depleted upstream glycolytic metabolites. **a** Western blot analysis showing the ability of DASA-58 (15 μM) and TEPP-46 (30 μM) in reducing TXNIP levels in the presence of the proteasome inhibitor MG132 (5 μM), whereas the presence of the translation inhibitor cycloheximide (10 μM) depleted TXNIP levels almost completely in MCF7 cells. **b** Western blot showing the ability of DASA-58 (15 μM) in reducing TXNIP levels in the absence of LKB1, STAT3, and AMPK. Predesigned siRNAs were used to achieve knock-down of LKB1, STAT3, and AMPK in MCF7 cells. **c**, **d** Western blot analysis showing the effects of ER signaling on TXNIP expression in the presence of DASA-58 (15 μM) in MCF7 cells. The ER blocker tamoxifen (5 μM) cannot rescue TXNIP levels in the presence of DASA-58 at short (upper panel) and long (bottom panel) time points (**c**). ER activation using E2 (100nM) also fails to rescue TXNIP expression in response to DASA-58 at short (upper panel) and long (bottom panel) time points (**d**). **e** Western blot showing TXNIP levels in MCF7 cells as triplicates in the presence of DASA-58 (15 μM, 72 h) alone or combined with the glycolytic inhibitor 2DG (5 mM, 24 h). Adding 2DG to the conditioned medium 24 h before the end of DASA-58 treatment period rescues TXNIP expression to the basal levels. **f** Schematic illustration representing the hypothesis that PKM2 activation leads to decreased upstream glycolytic metabolites leading to reduced TXNIP expression, an effect that can be counteracted with the glycolytic inhibitor 2DG

The monitored reduction in TXNIP expression was further investigated aiming to dissect the mechanism by which PKM2 activation leads to reduced TXNIP levels. To investigate TXNIP expression under PKM2 activation, MCF7 cells were treated with either DASA-58 or TEPP-46 for 72 h. Four hours before the end of the treatment period, MG132 (proteasome inh.) or CHX (translation inh.) was added to the conditioned medium. Figure 5a shows that MG132 treatment leads to accumulation of TXNIP that still can be counteracted by PKM2 activation. This data suggests that the reduction in TXNIP is not due to enhanced proteasomal degradation but rather lowered expression levels. AMPK signaling—which is as shown above activated in response to DASA-58 treatment—is reported to play a role in TXNIP degradation in response to energy stress allowing higher glucose flow. We used small interfering (si)RNA against AMPK, liver kinase B1 (LKB1), and the signal transducer and activator of transcription (STAT3), and neither of them proved to be of importance in the mechanism by which PKM2 activation is reducing TXNIP levels; as shown in Fig. 5b, DASA-58 can reduce TXNIP in the absence of AMPK, LKB1, and STAT3.

Due to the importance of ER signaling in cellular metabolism of BCa cells, the impact of PKM2 activation on TXNIP levels in MCF7 cells was challenged by the presence of either tamoxifen or estradiol. In the first set of experiments, MCF7 cells were treated with DASA-58 for 72 h, and tamoxifen (ER blocker) or estradiol (ER ligand) was added to the conditioned medium 0.5, 1, 2, 4, and 24 h before the end of the treatment. Figure 5c and d show that the presence of either ER modulators did not reverse the effect of DASA-58 on TXNIP expression, and PKM2 activation is still able to reduce TXNIP levels independently of ER signaling. In the second set of experiments, we co-treated the cells with DASA-58 combined with either tamoxifen or estradiol for 24, 48, and 72 h to test the effects of long-term ER signaling modulators. Figure 5c and d indicate that DASA-58 reduced TXNIP expression in the presence of tamoxifen or estradiol. In case of estradiol, reduced TXNIP expression was monitored 72 h after treatment which masked the reduction caused by DASA-58 treatment. TXNIP level is known to be subject to the availability to the upstream glycolytic intermediates, and thus, we used the glycolytic inhibitor 2DG to assess whether the reduced TXNIP levels are due to depleted upstream glycolytic metabolites resulting for the enhanced PK activity. The presence of the glycolytic inhibitor 2DG—as expected—leads to enhanced TXNIP expression (Fig. 5e) indicating elevated levels of upstream glycolytic metabolites. However, 24 h 2DG treatment rescued TXNIP levels in the presence of DASA-58 (72 h total).

The more general nature of this effect was validated in the prostate cancer cell line LnCap (supplementary figure S3). As shown above for the BCa cell line MCF7, DASA-58 treatment also strongly reduced TXNIP levels in the prostate cancer cell line LnCap, an effect that was also independent of TXNIP proteasomal degradation (supplementary figure S3).

Altogether, these data suggest that the PKM2 activation leads to depletion in TXNIP levels independent of AMPK and ER signaling, and not through enhanced proteasomal degradation but rather as a consequence of depleted upstream glycolytic intermediates.

### PKM2 activation could be exploited by other metabolic stressors

**Fig. 6:**
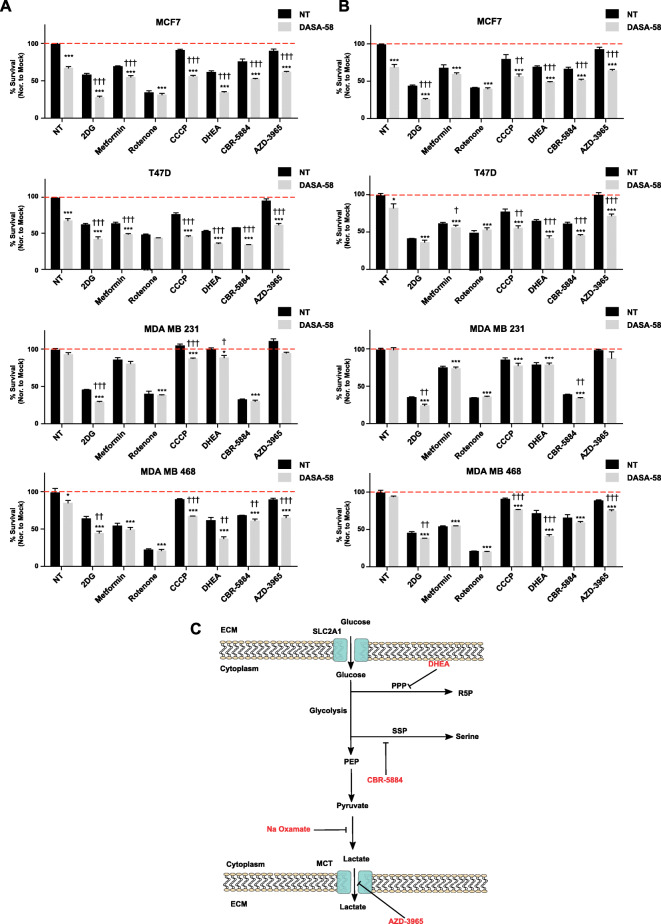
PKM2 activation potentiates the antitumor effects of other metabolic stressors. **a**, **b** Total protein staining using SRB assay (**a**) or metabolically active cells using MTT assay (**b**) showing the efficacy of combining DASA-58 (15 μM) with different metabolic stressors/inhibitors of glycolytic branching pathways in BCa cells. Data are presented as % survival normalized to the mock treatment and daggers (†) used to indicate statistical significance between the single and the combo treatment. **c** Schematic illustration representing the hypothesis of combining the PKM2 activator with other metabolic modulating drugs (red font)

We also sought to investigate the effects of PKM2 activation on the flow through the glycolysis’ branching pathways. Supplementary figure S4A shows that DASA-58 treatment does not alter the activity of G6PD (the first enzyme in the pentose phosphate pathway). With the latter one playing detrimental roles in regulating the cellular ROS scavenging ability [28], we measured intracellular ROS in response to DASA-58. As shown in supplementary figure S4B, the treatment does not seem to change ROS levels in MCF7 and T47-D under normal culture conditions. Nevertheless, challenging the system with hydrogen peroxide revealed that the treated cells are more susceptible to the treatment and more prone to accumulation of higher levels of ROS (supplementary figure S4B). PKM2 activity regulates serine synthesis with the latter one being a physiological activator of PKM2 [13]. Therefore, we investigated whether DASA-58 treatment affects cellular survival in the absence of serine. Supplementary figure S4C shows that DASA-58 does not affect cellular proliferation in the absence of serine. In order to investigate whether PKM2 pharmacological activation could be used as a potential anticancer strategy, we co-treated BCa cells with DASA-58 combined with different metabolic stressors. As shown in Fig. 6 a and b, among the studied combinations, the glycolytic inhibitor 2DG shows very promising effects when combined with DASA-58 in a panel of BCa cells. Furthermore, the mitochondrial uncoupler CCCP shows also promising effects in all the studied cell lines while metformin and rotenone (mitochondrial inhibitors) failed to show significant results in all the cell lines. Combining DASA-58 with inhibitors of either G6PD (DHEA) or PGDH (CBR-5884) is also capable of inducing the inhibitory effects of these drugs further indicating the importance of low PKM2 activity in controlling the flow through the upstream glycolytic intermediates (Fig. 6 a and b). Finally, due to the ability of DASA-58 to induce lactate production and secretion, combination with an inhibitor of MCT (AZD-3965) shows an enhanced antiproliferative activity in BCa cells (except for MDA MB 468) in the combination treatment (Fig. 6 a and b).

## Discussion

The low pyruvate kinase activity of PKM2 plays an essential role in supporting cancer altered metabolism through facilitating the rewiring of glycolytic intermediates into different anabolic pathways [20]. Different PKM2 pharmacological activators emerged and studied for their potential antitumor activities [20]. The study of Anastasiou et al., for example, showed reduced glucose uptake, lactate production, and reduction in many other important metabolite pools upon PKM2 pharmacological activation [20]. On the other hand, other reports showed a role of PKM2 activation in increasing lactate production and enhancing sensitivity to the glycolysis inhibitor 2-deoxyglucose (2DG) [21], as well as inducing serine auxotrophy [13]. The lack of PKM2 expression in benign tissue and the inability of the PKM2 activators to induce PK activity in the presence of other PK isotypes [20] make these drugs tempting to investigate aiming to open a new therapeutic window through targeting cancer metabolism [29]. The ability of PKM2 to shift between two conformations with distinct activities and its location at the end of glycolysis helps cancer cells to finely tune the faith of glucose depending on their needs [3, 30]. One option is mediated by elevated PKM2 activity leading to ADP phosphorylation, and the other option is mediated by the lower PKM2 activity allowing the upstream metabolites to be shunted into different anabolic pathways [8, 31]. However, another study showed that PKM2 may also facilitate an alternative pathway converting PEP into pyruvate without phosphorylating ADP and thus evading the inhibitory role of ATP on glycolysis [32]. In this report, we study the possible effects of PKM2 pharmacological activation on glucose rewiring and its impact on the energy homeostasis of breast cancer cells representing various molecular subtypes.

In our study, we show that PKM2 activation has minor anti-cancer effects on cell lines under normal culture conditions as others showed in their studies [20, 21]. Our data show that the ER + MCF7 cells are more prone to metabolic changes induced by the PKM2 allosteric activator DASA-58 than the other tested cell lines. The discrepancy between the level of PKM2 expression and its activity in the studied cells indicate a role of PKM2 post-translational modifications in controlling the enzyme’s glycolytic activity and/or propose that the presence of other PK isozymes contribute to the overall measured PK activity. This effect is clear when comparing the expression levels of PKM2 in MCF7 and MDA MB 231 and their corresponding activity levels. The differences in the basal levels of PKM2 together with different metabolic phenotypes/needs of the used different BCa cells dictated the cellular response to PKM2 activation. DASA-58 induces extracellular acidification levels and extracellular lactate concentration after longer incubation periods in all studied cell lines. Interestingly, lactate is not only a metabolic end product but emerged as an energy source [33], with reported ability of cancer cells to uptake lactate from culture medium for metabolic use [34]. It is also worth mentioning that the basal PK activity in some of the studied cell lines indicates that the efficacy of PKM2 activation is contextual and highly depends on the basal PK activity levels. DASA-58 and TEPP-46 also reduced oxygen consumption in the studied cells, an effect that was monitored when intact cells were treated with the molecule and not on isolated mouse liver mitochondria. Thus, we propose that the elevated PK activity when able to increase lactate production and secretion into the culture medium is also reducing the contribution of pyruvate into the TCA cycle and mitochondrial respiration. Pyruvate could be converted into lactate or metabolized by either pyruvate dehydrogenase of pyruvate carboxylase to enter the TCA cycle [35].

AMP-activated protein kinase is canonically responsible for cellular energy homeostasis which is activated in response to low AMP/ADP to ATP ratios through the upstream liver kinase B1 (LKB1) on T172 [16, 19]. AMPK, which when activated, can induce catabolism and inhibit anabolism aiming to rescue ATP levels [15, 16]. In our study, PKM2 activation causes an induction in AMPK phosphorylation and thus the phosphorylation and inhibition of ACC, indicating an energy crisis caused by the elevated PK activity. The reduction in ATP levels in response to DASA-58 treatment were minor however significant in case of MCF7—for example—and lead to AMPK activation. The cellular adaptation to the rewired glucose in response to DASA-58 treatment could be also sensed by AMPK as a nutrient-scarce condition leading to the phosphorylation of AMPK. Moreover, the DASA-58 contributed to further ATP depletion in the presence of the glycolytic inhibitor 2DG. These data together show that glucose rewiring by PKM2 activation reduces mitochondrial activity of MCF7 cells without damaging the mitochondria. Despite the activation in AMPK signaling, a known inducer of mitochondrial activity and biogenesis [36], PKM2 activation is still reducing mitochondrial activity. Another hypothesis is that the rewiring of glucose in response to PKM2 activation leads to reduction in TCA intermediates, thus, lowering mitochondrial activity which is reflected as an energy crisis leading to AMPK activation. We further show the importance of the relatively low activity of PKM2 in keeping AMPK in a low phosphorylation state, therefore, ensuring a high level of anabolic reactions in BCa cells. Due to the sensitivity of cancer cells to low energy state, AMPK signaling—even though controversial—was exploited as anticancer treatments [19]. The classic antidiabetic drug metformin for example was reported many times for its antitumor effects through activating AMPK signaling [17, 18, 37]. We exploited the ability of DASA-58 to induce AMPK signaling by showing the efficacy of combining it with metformin alongside other mitochondrial inhibitors that mostly showed enhanced AMPK phosphorylation and reduced survival. The metabolic vulnerability induced by DASA-58 treatment could be exploited, aiming to improve the anticancer effects of metabolism-altering drugs in future clinical trials.

Moreover, AMPK activation is reported to induce the degradation of thioredoxin-interacting protein (TXNIP) [38, 39]. TXNIP is induced in response to elevated upstream glycolytic intermediates, leading to inhibition of GLUT1 activity and thus glucose uptake [38, 40]. Two main mechanisms regulate TXNIP levels: (I) MondoA/MLX heterodimer transcription factor binds TXNIP promoter and increases its expression in response to elevated intracellular glucose [27, 41]. (II) AMPK activation leads to TXNIP proteasomal degradation allowing more cellular glucose uptake [38]. Our data implies that the monitored TXNIP reduction is independent of AMPK signaling. Furthermore, DASA-58 treatment in MCF7 cells elevates PK catalytic activity and thus depletes the upstream glycolytic metabolites which lead to TXNIP reduction. Even though TXNIP is reported to be a tumor suppressor [4, 42], TXNIP reduction mediated by DASA-58 treatment does not hamper the antitumor effects of other chemotherapeutics.

Altogether, our study shows that PKM2 pharmacological activation causes a multifaceted metabolic reprogramming in BCa cells. The elevated PK activity negatively regulated TXNIP expression independently of AMPK activation. TXNIP reduction reflects that PKM2 activation rewires intracellular glucose, leading to depleted upstream glycolytic intermediates and thus mimicking a nutrient-scarce condition even in the presence of high glucose levels in culture medium. The monitored effects render the cells more susceptible to metabolic challenges, an effect that could be exploited for potential usage of PKM2 activators in combination with other anticancer agents.

## Conclusions

In this study, we investigate the efficacy of PKM2 pharmacological activation on glucose metabolism in breast cancer cells. The investigated five breast cancer cell lines represent different molecular subtypes and show different expression levels of PKM2 and different levels of PK activity. These discrepancies in the PKM2 levels and PK activity are probably due to post-translational modifications (on PKM2) or to the expression of different PK isotypes. Nevertheless, we clearly show that cells expressing PKM2 but showing relatively lower PK activity (MCF7) are more sensitive to PKM2 activation and thus indicating a context-dependent response to PKM2 pharmacological activation. Furthermore, our data strongly indicate the roles of PKM2 activators in rewiring cellular glucose metabolism through enhanced lactate production and the subsequent reduced pyruvate submission to the TCA cycle leading to lower oxygen consumption and AMPK activation. Furthermore, the enhanced PKM2 activity was associated with reduction in TXNIP levels independent of AMPK activation. The ability of the glycolytic inhibitor 2DG to rescue TXNIP levels in the presence of DASA-58 indicates that the elevated PK activity is leading to depleted upstream glycolytic intermediates and thus reduction in TXNIP expression. Future studies to directly measure the availability of upstream glycolytic intermediates (i.e., glucose-6-phosphate) would be highly interesting and could further confirm our findings. Additionally, further characterization of the relation between TXNIP levels and PK activity could establish TXNIP as a marker for PK activity levels and thus the prediction of cellular response to PKM2 activation.

## Supplementary Information

**Additional file 1: Supplementary Figure S1.**. PKM2 activation Induces extracellular acidification levels in prostate cancer cells. Online measurement of extracellular acidification using pH sensors embedded on the bottom of 24 well-plates in MCF7 cells in reponse to DASA-58 (15 μM) or to TEPP-46 (30 μM).

**Additional file 2: Supplementary Figure S2.** PKM2 activation Induces phosphorylation levels of ACC in prostate cancer cells. Western blot analysis showing tACC/pACC (S79) levels in a panel of prostate cancer cell lines in response to either DASA-58 (15 μM) or TEPP-46 (30 μM). Treatments performed in triplicates and vinculin is used as a loading control.

**Additional file 3: Supplementary Figure S3.**. PKM2 activation reduces TXNIP levels in LnCap cells. Western blot analysis showing TXNIP levels in LnCap cells in response to either DASA-58 (15 μM) alone (upper panel) or combined with the proteasome inhibitor MG132 or the translational inhibitor CHX (lower panel). Vinculin is used as a loading control.

**Additional file 4: Supplementary Figure S4.** PKM2 activation does not change G6PD activity . (A) G6PD activity in crude extract of BCa cells in response to DASA-58 (15μM), data presented as absorbance values at 340 nm resulting from the buildup of NADPH. (B) intracellular ROS levels in MCF7 and T47-D cells in response to either DASA-58 (15μM) alone or followed by 2 h of H2O2 (200 μM) treatment. Daggers (†) are used to indicate statistical significance between the single and combo treatment. (C) Total protein staining using SRB assay showing the relative survival of MCF7 cells in response to DASA-58 (15 μM) in the absence of serine. Data are presented as % survival normalized to the mock treatment.

## Data Availability

All data generated or analyzed during this study are included in this published article.

## Abbreviations

PKM2: Pyruvate kinase M2
AMPK: 5' AMP-activated protein kinase
TXNIP: Thioredoxin-interacting protein
BCa: Breast cancer
GLUT1: Glucose transporter 1
PEP: Phosphoenolpyruvate
2DG: 2-deoxyglucose
ER: Estrogen receptor
LKB1: Liver kinase B1
STAT3: Signal transducer and activator of transcription 3
MMP: Mitochondrial inner membrane potential
