# Supplementary material for: Pharmacological activation of pyruvate kinase M2 reprograms glycolysis leading to TXNIP depletion and AMPK activation in breast cancer cells
Source: Cancer Metab. 2021 Jan 22;9:5. doi: 10.1186/s40170-021-00239-8 (PMC7821649; doi:10.1186/s40170-021-00239-8)
Supplement: Supplementary file 1 — Additional file 1: Supplementary Figure S1.. PKM2 activation Induces extracellular acidification levels in prostate cancer cells. Online measurement of extracellular acidification using pH sensors embedded on the bottom of 24 well-plates in MCF7 cells in reponse to DASA-58 (15 μM) or to TEPP-46 (30 μM). [file 40170_2021_239_MOESM1_ESM.docx]

**Supplementary Figure S1 PKM2 activation Induces extracellular acidification levels in prostate cancer cells.** Online measurement of extracellular acidification using pH sensors embedded on the bottom of 24 well-plates in MCF7 cells in reponse to DASA-58 (15µM) or to TEPP-46 (30µM).
