# Supplementary material for: Pharmacological activation of pyruvate kinase M2 reprograms glycolysis leading to TXNIP depletion and AMPK activation in breast cancer cells
Source: Cancer Metab. 2021 Jan 22;9:5. doi: 10.1186/s40170-021-00239-8 (PMC7821649; doi:10.1186/s40170-021-00239-8)
Supplement: Supplementary file 2 — Additional file 2: Supplementary Figure S2. PKM2 activation Induces phosphorylation levels of ACC in prostate cancer cells. Western blot analysis showing tACC/pACC (S79) levels in a panel of prostate cancer cell lines in response to either DASA-58 (15 μM) or TEPP-46 (30 μM). Treatments performed in triplicates and vinculin is used as a loading control. [file 40170_2021_239_MOESM2_ESM.docx]

**Supplementary Figure S2 PKM2 activation Induces phosphorylation levels of ACC in prostate cancer cells.** Western blot analysis showing tACC/pACC (S79) levels in a panel of prostate cancer cell lines in response to either DASA-58 (15µM) or TEPP-46 (30µM). Treatments performed in triplicates and vinculin is used as a loading control.
