# Supplementary material for: Pharmacological activation of pyruvate kinase M2 reprograms glycolysis leading to TXNIP depletion and AMPK activation in breast cancer cells
Source: Cancer Metab. 2021 Jan 22;9:5. doi: 10.1186/s40170-021-00239-8 (PMC7821649; doi:10.1186/s40170-021-00239-8)
Supplement: Supplementary file 3 — Additional file 3: Supplementary Figure S3.. PKM2 activation reduces TXNIP levels in LnCap cells. Western blot analysis showing TXNIP levels in LnCap cells in response to either DASA-58 (15 μM) alone (upper panel) or combined with the proteasome inhibitor MG132 or the translational inhibitor CHX (lower panel). Vinculin is used as a loading control. [file 40170_2021_239_MOESM3_ESM.docx]

**Supplementary Figure S3 PKM2 activation reduces TXNIP levels in LnCap cells.** Western blot analysis showing TXNIP levels in LnCap cells in response to either DASA-58 (15µM) alone (upper panel) or combined with the proteasome inhibitor MG132 or the translational inhibitor CHX (lower panel). Vinculin is used as a loading control.
