# Supplementary material for: Pharmacological activation of pyruvate kinase M2 reprograms glycolysis leading to TXNIP depletion and AMPK activation in breast cancer cells
Source: Cancer Metab. 2021 Jan 22;9:5. doi: 10.1186/s40170-021-00239-8 (PMC7821649; doi:10.1186/s40170-021-00239-8)
Supplement: Supplementary file 4 — Additional file 4: Supplementary Figure S4. PKM2 activation does not change G6PD activity . (A) G6PD activity in crude extract of BCa cells in response to DASA-58 (15μM), data presented as absorbance values at 340 nm resulting from the buildup of NADPH. (B) intracellular ROS levels in MCF7 and T47-D cells in response to either DASA-58 (15μM) alone or followed by 2 h of H2O2 (200 μM) treatment. Daggers (†) are used to indicate statistical significance between the single and combo treatment. (C) Total protein staining using SRB assay showing the relative survival of MCF7 cells in response to DASA-58 (15 μM) in the absence of serine. Data are presented as % survival normalized to the mock treatment. [file 40170_2021_239_MOESM4_ESM.docx]

**Supplementary Figure S4 PKM2 activation does not change G6PD activity .** (A) G6PD activity in crude extract of BCa cells in response to DASA-58 (15µM), data presented as absorbance values at 340 nm resulting from the buildup of NADPH. (B) intracellular ROS levels in MCF7 and T47-D cells in response to either DASA-58 (15µM) alone or followed by 2h of H2O2 (200 µM) treatment. Daggers (†) are used to indicate statistical significance between the single and combo treatment. (C) Total protein staining using SRB assay showing the relative survival of MCF7 cells in response to DASA-58 (15 µM) in the absence of serine.
